# Insights into APC/C: from cellular function to diseases and therapeutics

**DOI:** 10.1186/s13008-016-0021-6

**Published:** 2016-07-13

**Authors:** Zhuan Zhou, Mingjing He, Anil A. Shah, Yong Wan

**Affiliations:** Department of Cell Biology, University of Pittsburgh School of Medicine and University of Pittsburgh Cancer Institute, 5117 Centre Avenue, Hillman Cancer Center, HCC2.6c, Pittsburgh, PA 15213 USA; State Key Laboratory of Oral Diseases, West China Hospital of Stomatology, Sichuan University, Chengdu, 610041 Sichuan People’s Republic of China

**Keywords:** Anaphase-promoting complex/cyclosome, Ubiquitylation, Cell cycle, Genome stability, Neuron, Stem cell, Metabolism, Autophagy, Apoptosis and senescence, Tumorigenesis, Anti-cancer treatment

## Abstract

Anaphase-promoting complex/cyclosome (APC/C) is a multifunctional ubiquitin-protein ligase that targets different substrates for ubiquitylation and therefore regulates a variety of cellular processes such as cell division, differentiation, genome stability, energy metabolism, cell death, autophagy as well as carcinogenesis. Activity of APC/C is principally governed by two WD-40 domain proteins, Cdc20 and Cdh1, in and beyond cell cycle. In the past decade, the results based on numerous biochemical, 3D structural, mouse genetic and small molecule inhibitor studies have largely attracted our attention into the emerging role of APC/C and its regulation in biological function, human diseases and potential therapeutics. This review will aim to summarize some recently reported insights into APC/C in regulating cellular function, connection of its dysfunction with human diseases and its implication of therapeutics.

## Background

The ubiquitin–proteasome system (UPS) plays a critical role in regulating numerous cellular pathways through controlling the abundance, activity and localization of an enormous variety of cellular proteins [1]. Overall, three essential enzymes, E1, E2, and E3, the activating, conjugating, and ligase enzymes respectively that methodically relocate Ubiquitin molecules [2]. The ubiquitin chain attached substrate protein will be then either recognized by the proteasome for destruction or undergo for modification [3–5]. Specifically, the E3 ligase can be classified into the HECT (homologous to the E6-AP carboxyl terminus) domain containing E3s and the Really Interesting New Gene (RING) domain containing E3s [6].

It is thought that HECT E3s usually catalyze the formation covalent bonds between cysteine residues of ubiquitin molecules before transferring the ubiquitin molecule to the protein, whereas RING E3s catalyze the transfer of the ubiquitin from the E2 to the substrate protein [5]. The modification of substrate protein by ubiquitin molecule could be through mono-ubiquitylation or various types of poly-ubiquitylation [2]. It has been demonstrated that seven individual lysine residues on the ubiquitin molecule, including K6, K11, K27, K29, K33, K48 and K63, could form different types of ubiquitin chain attaching to the substrate protein in order to achieve various physiological regulation [7–9]. Results from the human genome sequencing indicated the presence of approximately 600 different ubiquitin ligases [5]. Among these E3 ligases, the Skp1–Cullin-1–F-box protein (SCF) and APC/C, are two well studied RING finger type E3 ligases, which provide us good example to dissect other new E3 ligases [5, 10, 11]. This review will focus on some relatively new aspects of APC/C reported in recent years in cell cycle control, apoptosis, energy metabolism, autophagy, and carcinogenesis and drug development.

## Architecture of APC/C

**Fig. 1:**
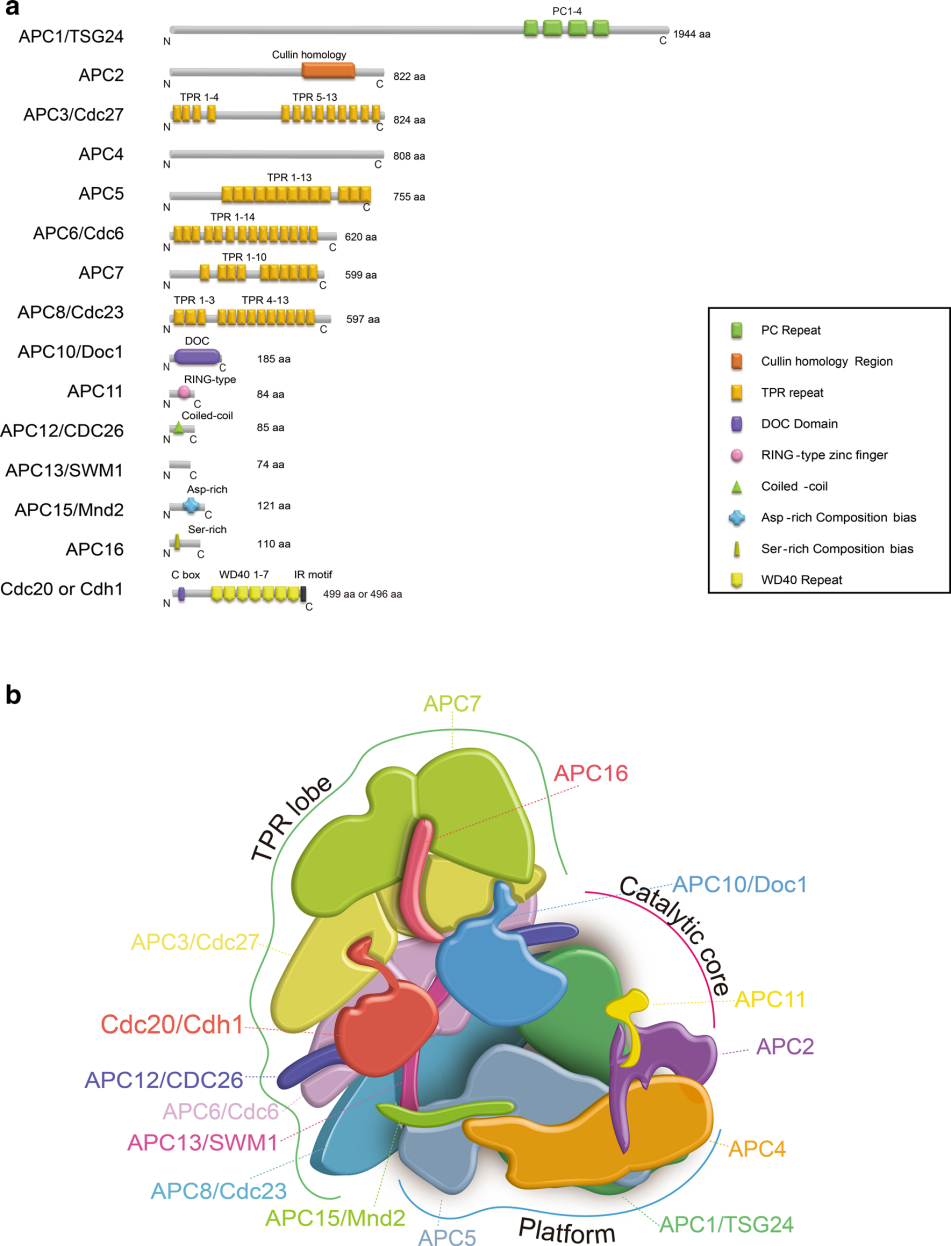
Structure and genetics characteristics of APC/C. **a** Graphic representation of human (*Homo sapiens*) APC/C subunits. All domains are signified by colored boxes and full length protein sequences are shown by *gray lines*. **b** A schematic illustration of the structure organization of APC/C complex. APC/C complex contains three sub-complexes: the scaffolding platform, the TPR lobe and the catalytic core. The scaffolding platform consists of APC1, APC4 and APC5. The catalytic core consists of APC2 (Cullin family related protein), APC10, APC11 (RING finger protein), Cdc20 or Cdh1 (catalytic coactivators) and substrate. The TPR lobe consists of APC3, APC6, APC8, APC7, APC13, APC16, and Cdc26. The scaffolding platform connects the TPR lobe to the catalytic core

In comparison with SCF complex, APC appears to be more sophisticated in term of its architecture due to the feature of its large complex [10–12]. The APC/C is a 1.5 megadaltons assembly ubiquitin ligase complex comprising 19 subunits [13, 14]. It takes relatively long to biochemically dissect and recapitulate this multiple-subunit enzyme. Now it is clear, in vertebrates, the APC/C holoenzyme is a complex of 15 different proteins including ANAPC1/APC1/TSG24, ANAPC2/APC2, ANAPC3/APC3/Cdc27, ANAPC4/APC4, ANAPC5/APC5, ANAPC6/APC6/Cdc6, ANAPC7/APC7, ANAPC8/APC8/Cdc23, ANAPC10/APC10/Doc1, ANAPC11/APC11, ANAPC12/APC12/CDC26, ANAPC14/APC13/SWM1, ANAPC15/APC15/Mnd2, ANAPC16/APC16, as well as the co-activator subunit (Cdc20 or Cdh1) [13, 15, 16]. The schematic representation of the conserved domain of these subunits was shown in Fig. 1a.

Comprehensive studies with structural, genetic and biochemical approaches have sketched the general architecture and revealed the underlying mechanism by which APC/C recognizes, and catalyzes ubiquitination of the targeting proteins [13, 16–18]. Overall, the APC/C complex consists of three sub-complexes: the scaffolding subcomplex platform, the catalytic and substrate identification core and a tetratricopeptide repeat (TPR) arm (Fig. 1b). The scaffolding sub-complex is composed of APC1/TSG24, APC4 and APC5; the catalytic sub-complex contains APC2, APC10 and RING finger protein APC11; and the TPR arm consisting of APC3, APC6, APC7 and APC8, which provides binding sites for the scaffolding subunit and one of the coactivators (Cdc20 or Cdh1). On the platform, the APC1 subunit is the bridge between the catalytic portion and the TPR arm. APC3, in the catalytic sub-complex acts as a platform for the catalytic core. In turn, APC11 regulates the interface with E2 enzymes. It is also worth noting that APC10 forms a majority of where the substrate binds. The APC10 might also contribute to substrate recruitment via its degron-recognition module [16]. The TPR arm functions as the important scaffolding to the APC/C. In addition to APC3, APC6, APC7 and APC8 in the TPR arm, other factors including APC12, APC13, and APC16 may also play a role in stabilizing the TPR arm. APC12, APC8, APC7, APC6 and APC3 are present as dimers, other subunits exist as monomers. The TPR motifs of APC3 recruit Cdc20 or Cdh1 via binding to homologous carboxyl (C)-terminal Ile-Arg sequences displayed at APC10 and Cdc20 or Cdh1. Interestingly, the Barford group has recently demonstrated atomic structures of APC/C–coactivator complexes with either an UbcH10–ubiquitin conjugate or Emi1 via cryo-electron microscopy. By analysis of these structures, it was shown how Emi1 antagonizes the two E2s, UbcH10 and Ube2S and details of the initiating sequential ubiquitination reaction [13]. While our understanding of APC/C for its enzymology and 3D architecture has been tremendously expanded, some important knowledge about APC is still missing, for instance, the assembly mechanism of APC/C under various cellular conditions and if all 15 subunits are necessary for APC/C acting under different physiological circumstances.

## Mechanisms of ubiquitin chain formation by the APC/C

**Fig. 2:**
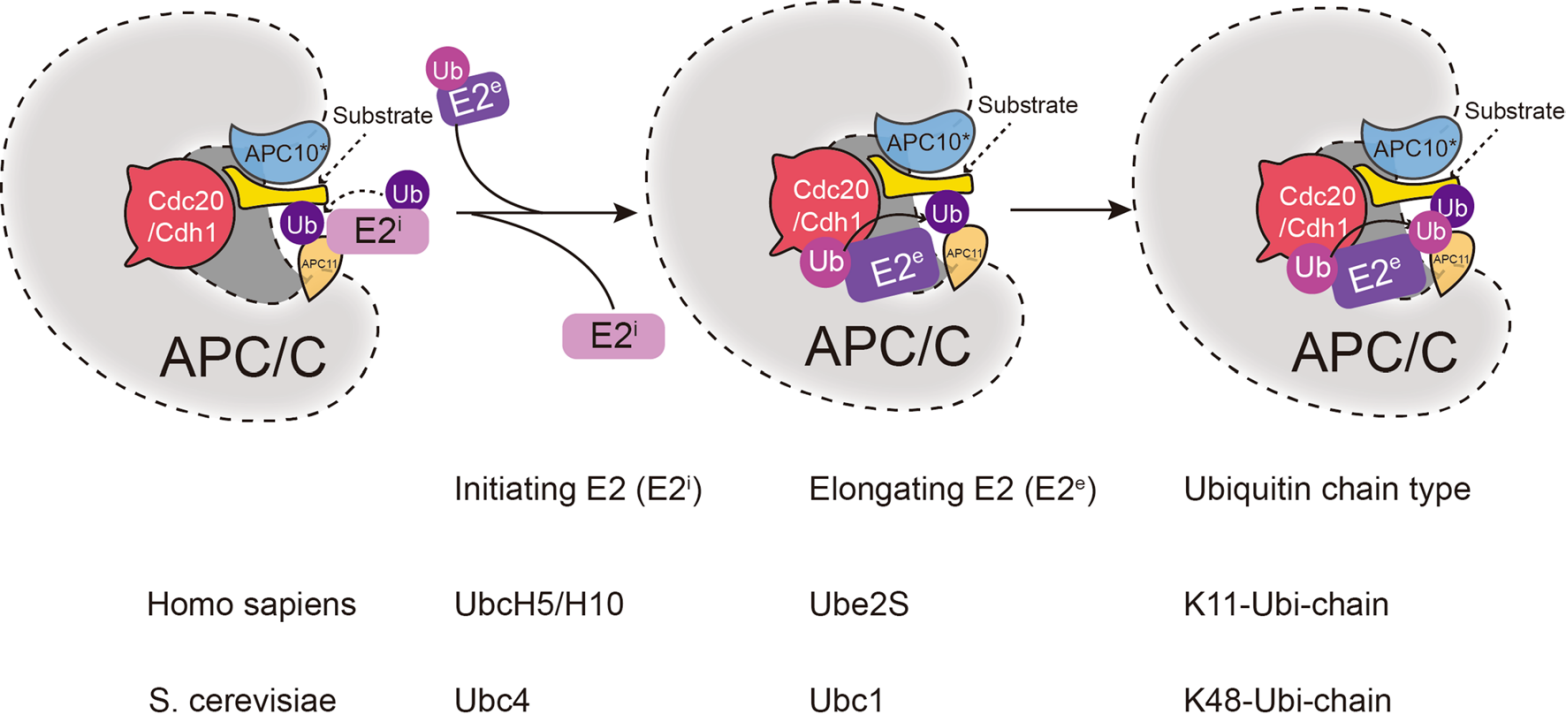
Mechanisms of Ubiquitin chain formation by the APC/C. The APC/C catalytic polyubiquitination chain formation by two-step sequential reactions with two E2s: initiating E2 (E2^i^) and elongating E2 (E2^e^). In *homo sapiens*, the “initiating” E2s, UBCH5 or UBCH10, with APC/C complex ligates ubiquitin to substrate and the “elongating” E2 UBE2S expands a K11 polyubiquitination chain on the Ubiquitin-primed substrate. The UBCH5 or UBCH10 binds to APC11 to initiate substrate ubiquitinated, then the UBE2S is recruited to the APC/C by Cdh1/Cdc20. In the chain elongating assembly, the APC/C binding site and the non-canonical distinct APC11 RING surface helps UBE2S deliver K11 polyubiquitination chain to the substrate. In *S. cerevisiae,* the “initiating” E2, Ubc4 and the “elongating” E2 Ubc1 deliver a K48 polyubiquitination chain to the substrate

Polyubiquitination by E1, E2, and E3 enzyme cascade is a principal mechanism modifying protein function. APC complex catalyzes polyubiquitination by two-step sequential reactions with two different E2s [19]. In studies done in *S. cerevisiae*, it was shown that the APC/C generates lysine 48 (K48)-linked chains and that two different E2s, Ubc4 and Ubc1, regulate the extension [17]. The initial modification of K48 is accomplished by Ubc4 and Ubc1 is responsible for chain elongation [20]. Making of K48-connected chains requires residues situated in two loops in the region of the active site cysteine of Ubc1 [21]. Ubc4 and Ubc1 having the conserved scaffold have developed distinctive mechanisms to perform the same work that generating K48-linked poly-Ubiquitin chains. In human, the “initiating” E2s, UBCH10 or UBCH5 (homolog of *S. cerevisiae* Ubc4, including UbcH5a and UbcH5c), with APC/C complex ligates ubiquitin to Cdc20 or Cdh1-bound substrate. The “elongating” E2 UBE2S extends a poly-Ub chain on the Ub-prepared substrate. UbcH5a and UbcH5c can utilize ubi-K11, ubi-K48, and ubi-K63 to catalyze the ubiquitination of APC/C–Cdh1-substrates, however, UbcH10 only catalyzes chains linked via K11, which is different in S. cerevisiae [22] (Fig. 2).

In recent studies in humans, there were two E2 enzymes identified, UBCH10 and UBE2S that were found to be crucial regulators of cell division and identified as potential signalers for the degradation of APC/C. It was also found in recent studies that chains of K11 are increasingly upregulated in mitotically active cells where substrates of APC/C are degraded [23]. It was shown that linkages of K11 by the E2, UBE2S, promotes the degradation of APC/C substrates independently of K48 chains [24]. K11 chains which triggering degradation of a variety of cell cycle regulators during mitosis are dispensable for most APC/C substrates [4].

Recently studies revealed how E2 enzymes UBCH10 and UBE2S control K11 chain initiating and elongating. Firstly UBCH10 an E2 enzyme is recruited to APC/C and subsequently stimulated for ubiquitination. It is then situated for substrate targeting via complexing with the APC cullin-RING core and interactions with APC2. An interaction between the UBC domain and the Apc11 RING surface activates UbcH10, which subsequently triggers an E2-ubiquitin intermediary for substrate alteration. By means of KEN- and D-box binding to Cdh1 and the APC core, the E3 primed substrate is co-recruited at a separation. The APC/C–Cdh1–UBCH10∼Ub–substrate complex intermediately ligates Ub directly to an E3 primed substrate [25]. Since UbcH10 and Apc11-RING communicate via a classic E2-RING interface, the APC/C has been shown to stimulate the innate catalytic activity of UbcH10–ubiquitin by fortifying a closed conformation state that resulting the lysine on the substrate attacking the E2-ubiquitin thioester bond and transferring of ubiquitin. Subsequently, there is poly-Ub chain elongation in which ubiquitin of the current chain capacities as the acceptor as well as the substrate. Cdc20 or Cdh1, recognizes UBE2S by means of its particular C-terminal locale and exchanges the ubiquitin to APC/C. In this reaction, APC/C determines the binding location for both acceptor ubiquitin and the E2 (UBE2S) enzyme, whereas the RING domain of APC11, which is required to position the acceptor ubiquitin, seems to be responsible for acceptor recognition [19, 26]. It has also been shown that during the creation of the chain, the discrete APC11 RING surface aids in delivering the Ubiquitin primed substrate to accept an additional ubiquitin from UBE2S. Therefore, UBCH10 and UBE2S have comparable affinities to APC/C. The sequential binding of UBCH10 and UBE2S are caused by binding to distinct sites on the complex. Thus, APC/C and specific adaptor proteins need to distinguish different substrates as well as ubiquitin molecules for the first ubiquitylation. For subsequent chain elongation, both ubiquitin-charged UbcH5/UbcH10 and UBE2S are required (Fig. 2).

The APC/C inhibitor protein early mitotic inhibitor 1 (Emi1) antagonizes the function of UbcH10 and UBE2S, which are accountable for catalyzing chain origination and elongation, correspondingly [13]. The mechanism of action of Emi1 is that the zinc-binding region (ZBR) identifies D-box motifs on the different substrate and subsequently inhibits the UbcH10-dependent APC/C activity. The architecture of ZBR motif named in-between-RING (IBR) associated with Apc2–Apc11 and the linker between Emi1 D box and ZBR motif shapes an α-helix that complexes against the Emi1-ZBR β-sheet and docks onto the site on Apc11-RING where UbcH10 binding. It is worth noting that the Emi1 ZBR motif does not inhibit the UBE2S-catalyzed ubiquitination reactions [27, 28]. The C-terminal LRRL motif of Emil1 is indistinguishable to the LRRL motif on UBE2S, which is required for APC/C–UBE2S binding. Thus, the Emil1 antagonizes UBE2S binds to APC/C via LRRL motif and inhibits APC/C activity.

## Regulations of APC/C

**Fig. 3:**
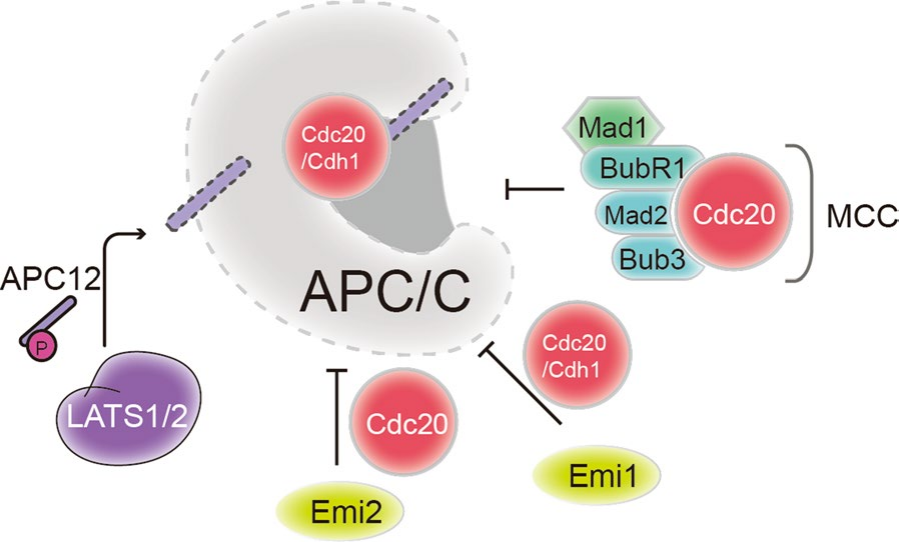
Regulation of APC/C activity. The APC/C activity is governed by catalytic coactivators Cdh1/Cdc20, mitotic checkpoint complex (MCC) and Emi1/2. The MCC complex including MAD2/MAD3, BUB3, BubR1 and Cdc20 generates an inhibitor of APC/C activity to prevent premature anaphase onset. The MCC is able to inhibit both unliganded Cdc20 and Cdc20 bound to the APC/C. Emi1 functions as an APC/C “pseudo-substrate” to block APC/C’s access to other substrates, whereas Emi2 inhibits APC/C by targeting its association with the coactivator Cdc20. The posttranslational modifications, including phosphorylation, could modulate APC/C activity. The large tumor suppressor kinase 1 and 2 (LATS1/LATS2) phosphorylate APC12/CDC26 to modulate TPR lobe assembly and APC/C activity

It has been demonstrated that regulation of APC turns to be sophisticated by various mechanisms such as substrate specific factors and different types of posttranslational modifications, including phosphorylation, sumoylation and acetylation [12, 29–31]. Principally, the activation of APC/C is governed by two WD-40 family proteins Cdh1 or Cdc20 [12]. Despite these two substrate factors, the on/off of APC during the cell cycle progression is also determined by phosphorylation, the mitotic checkpoint complex (MCC) and interphase early mitotic inhibitor 1 (Emi1) (Fig. 3).

Activation of APC/C by Cdc20 or Cdh1 has been thoroughly studied in the past 20 years. C termini on both Cdc20 and Cdh1 include a WD40 domain that attracts different APC/C substrates along with promoting ubiquitylation via augmenting the interaction of APC/C and UbcH10 and UBE2S (Fig. 1a) [32]. It is also proposed that Cdc20 and Cdh1 bind to different regions of APC8 and APC3 via interactions with the TPR motifs (Fig. 1b) [32]. Despite Cdc20 and Cdh1 have the similar structures, they activate the APC/C at distinctive periods. Cdc20 associates with APC/C in early mitosis which is followed by the destruction of different substrates involved in mitosis. Subsequently, Cdh1 replaces Cdc20 amid anaphase and also extending into the G1 phase. CDK1 subsequently phosphorylates Cdh1, which then has the effect of inhibiting its interaction with APC/C until later in anaphase. The diminishing CDK1 activity coupled with expanded phosphatase activity is subsequently followed by Cdh1 dephosphorylation, which then ties to and initiates the APC/C activity, in this way bringing on substrate degradation in late mitosis and during G1 phase.

Cdc20 and Cdh1 are well-established substrate receptors for APC/C. Recently study showed that these two adaptors can also target cell cycle proteins for destruction through a second ubiquitin ligase, Parkin [33]. Parkin networks with the APC/C coactivators Cdc20 and Cdh1, which is independent of the APC/C–Cdh1/Cdc20 complex to degrade some key mitotic controllers such as Cyclin B1, Aurora-B. Parkin insufficiency leads to its substrates aberrant expression, mitotic imperfections, genome instability as well as tumorigenesis. Cdh1 and Cdc20 both could be acetylated and their hyperacetylation inhibits the APC/C activity. The sirtuin family member SIRT2 could catalyze Cdh1 and Cdc20 deacetylation to maintain normal mitosis. SIRT2 deficiency also leads to mitotic defects, genome instability, as well as tumorigenesis [34].

The mitotic checkpoint complex (MCC), which contains spindle assembly checkpoint (SAC) proteins including MAD2/MAD3 (mitotic arrest deficient), BUB3 (budding uninhibited by benzimidazole), and Cdc20, prevents immature anaphase onset through APC/C activity inhibition [35]. The inhibition regulation of APC/C activity by MCC is fundamental for the SAC. The MCC components have the action of inhibiting the attraction of different mitotic intermediates, such as cyclin B and securin, which require recognition via KEN-box and D-box motifs, and consequently restrain APC/C robustly ubiquitinates diverse substrates. The MCC is able to inhibit an additional Cdc20 that has previously interacted with APC/C, which has the action of preventing anaphase step in the absence of kinetochore signaling [36]. Bub3 kinetochore localization is needed for the correct time signaling of anaphase commencement and for usual associated with APC/C and Cdc20 [37]. The BUBR1 activation regulated by Bub3 has two distinct roles: in unattached kinetochores, Bub3 enhances signaling to form BubR1 and Cdc20 complex via inherent binding sites downstream of kinetochore-produced complexes, thus promoting two specific BubR1-Cdc20 binding interactions. Cdc20–Mad2 binding lead to exposing the binding site of Cdc20 for BubR1 binding via its N-terminal conserved Cdc20 binding domain, whereas the Bub3 promotes Cdc20-BubR1 binding [38]. Recently studies demonstrated that Bub3–BubR1-dependent appropriation of Cdc20 at DNA breaks could facilitate proper segregation of broken chromosomes [39].

Early mitotic inhibitor 1 (Emi1) has a significant role during interphase of the cell cycle to constrain APC/C activity, which is the subsequent consequence of increasing the levels of mitotic cyclins for entry into mitosis. Emi1 contains a Zn-Binding Region (ZBR) and a conserved D-box, both of which contribute to the inhibition of APC/C activity through binding to the APC/C core complex and its coactivators Cdc20 or Cdh1. Emi1 binds APC/C coactivators via its D-box with high affinity, preventing the recruitment of APC/C substrates to the APC/C core complex, while the ZBR domain directly suppresses APC/C E3 ligase activity by associating with the APC/C core subunits. In this scenario, Emi1 functions as an APC/C “pseudo-substrate” to block APC/C’s access to other substrates [40]. Phosphorylation by Plk1 and ubiquitination by SCF/β-TrCP (β-transducin repeat-containing protein) at the onset of mitosis leads to Emi1 degradation, which resulting APC/C activation. In addition to Emi1, Emi2 likewise hinders APC/C activity by competitively inhibiting association of E2 Ube2S with APC10 subunit of the APC/C [41].

Phosphorylation regulation the subunits of APC/C is crucial for APC/C activity modulation. Recent study revealed several novel phosphorylation regulation the subunits of APC/C. The large tumor suppressor kinase 1 and 2 (LATS1/LATS2) are serine/threonine kinases of the AGC kinase family and core components of the Hippo pathway in mammals. APC12/CDC26 is phosphorylated by LATS 1 and LATS2 to modify the interactions of the tetratricopeptide repeat subcomplex of APC/C and to subsequently regulate its activity [42].

During mitosis, there have been studies that show regulating translation of proteins rather than transcription of mRNA that is the most significant mechanism regulating protein expression during mitosis. One of the most pronounced translationally-repressed genes is Emi1 [43]. The translational repression of Emi1 is required for full APC activation. Therefore, gene-specific translational repression may complement post-translational mechanisms for regulating APC/C activity.

The deubiquitinases (DUBs) are apparatuses of the ubiquitin proteasome system that catalyze the elimination of ubiquitin molecules from proteins causing distorted signaling in protein stability [44, 45]. Nearly 100 DUBs are encoded by the human genome to work in concert with E3 ligases [46]. Several deubiquitinases have been reported to counteract APC/C. Ubiquitin-specific protease 44 (USP44) works by deubiquitinating Cdc20 and promote the MAD2-Cdc20 complex stabilization [47]. During cell cycle, deubiquitinase USP37 protein and activity are fluctuated: in G1 phase, the E2F transcription factors trigger its expression; and then its protein levels accumulated in G1/S; following phosphorylated by CDK2, its activity reach to peak; finally its protein was degraded in late mitosis. In G1/S, Activated USP37 binds to Cdh1 and deubiquitinates cyclin A, which Promote S Phase Entry [48]. Deubiquitinase USP22 is a substrate of APC/C–Cdc20 during cell exit from M phase. USP22 is activated by CDK1 phosphorylation and deubiquitinates and stabilizes Cyclin B1 to promote cell cycle progression [49]. In budding yeast, the deubiquitinase Ubp15 associates Cdh1 and S-phase cyclin gene Clb5. Clb5 is proteolyzed by APC/C and is deubiquitinated by Ubp15. Accumulated Clb5 by Ubp15 deubiquitinating activity is critical for timely entry into S phase [50]. Therefore, the tango between APC and deubiquitinase contributes to the generation of the switch-like transition controlling cell cycle progression [51].

## APC/C in cell cycle regulation

**Fig. 4:**
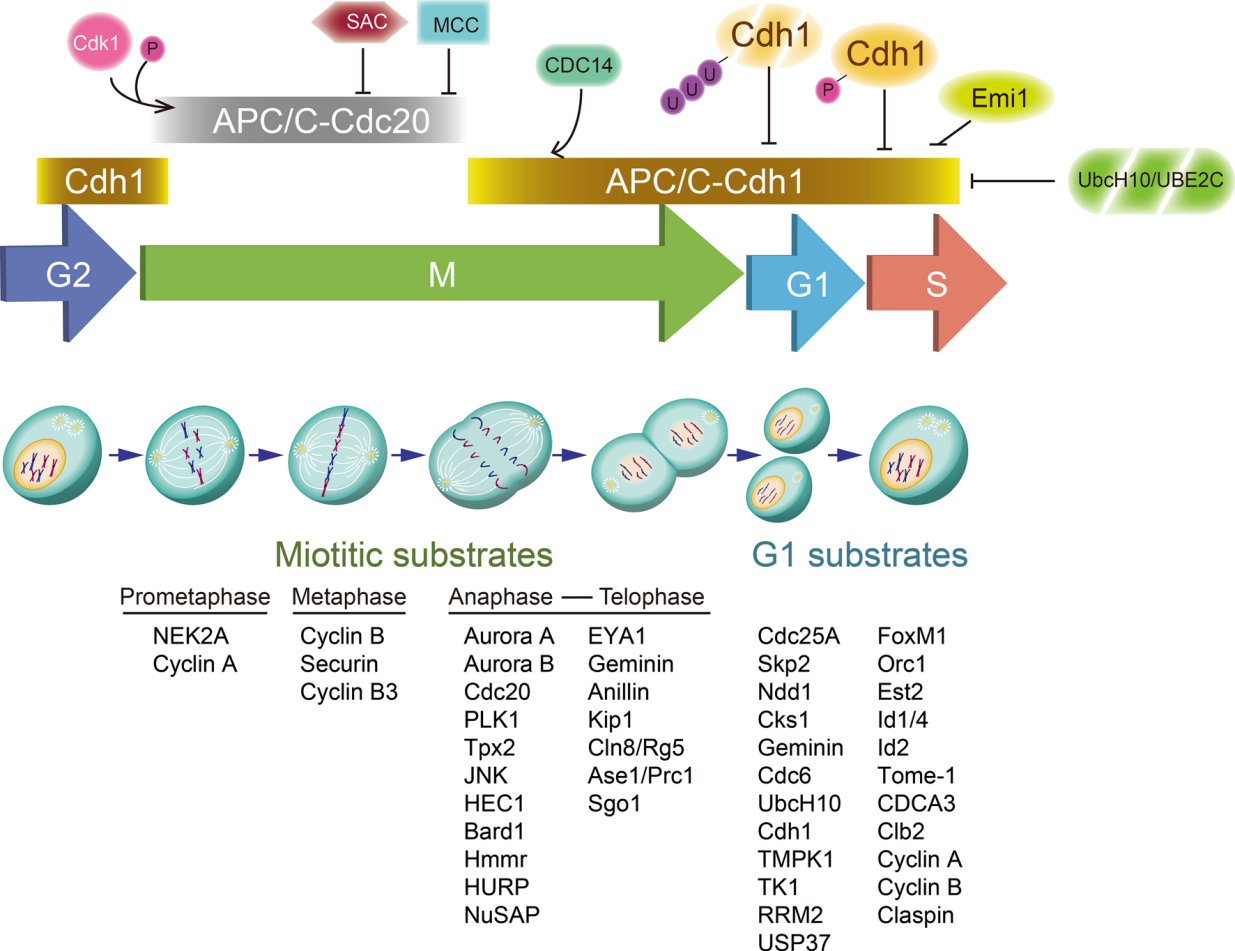
APC/C in cell cycle regulation. The regulation of APC/C activity and the order degradation of APC/C substrates during cell cycle progression. Regulation of cell cycle progression by the APC/C occurs primarily through the temporal coordination of Cdc20 or Cdh1. APC/C–Cdc20 degrades substrates in early and mid-mitosis, while APC/C–Cdh1 degrades substrates after anaphase commencement, during the end of mitosis and G1 phase. During G2/M transition phase, APC/C–Cdc20 is activated by CDK1 phosphorylation, whereas is inhibited by spindle assembly checkpoint (SAC) and mitotic checkpoint complex (MCC). When checkpoint requirement is satisfied, APC/C–Cdc20 ubiquitylates Cyclin-A, NEK2A in prometaphase and securin and cyclin B1 in metaphase. When cell commences to anaphase, Cdh1 is dephosphorylated by CDC14 and activates APC/C–Cdh1. During anaphase and telophase, APC/C–Cdh1 ubiquitylates substrates including Cdc20, Aurora kinases, PLK1, TPX2, spindle-binding proteins and stress-activated kinases. During G1 phase, APC/C–Cdh1 degrades mitotic cyclins such as Cdc25A, Skp2. During G1/S transition and G2 phase, APC/C–Cdh1 is inactivated by Emi1, Cdh1 degradation, phosphorylation by Cyclin A/Cdk2 and degradation of E2s

The impact of the APC/C function was initially implicated in the regulation of cell cycle progression, although now it is well known for its multifunctional role in the regulation of genomic stability, apoptosis, metabolism and development through degradation of specific functional proteins. Regulation of cell cycle advancement by the APC/C occurs primarily through the temporal coordination of two co-activators, Cdc20 or Cdh1, which form either the APC/C–Cdc20 or APC/C–Cdh1 E3 ligase complex. Although APC/C–Cdc20 or APC/C–Cdh1 have some substrates overlap, APC/C–Cdc20 primarily controls the metaphase to anaphase shift and mitotic exit, while APC/C–Cdh1 is primarily active during the end of mitotic exit and early G1 phase (Fig. 4).

Cdc20 but not Cdh1 plays an important role in regulating G2 progression. During G2 phase, Cdc20 is phosphorylated by Cdk1 and other mitotic kinases, which activates APC/C–Cdc20 in part by promoting the interaction between Cdc20 and the APC/C core complex [52, 53]. The SAC activates and sequester Cdc20 from the APC/C core complex by the mitotic checkpoint complex (MCC) when occurring aberrant mitotic events, such as misaligned spindles or improperly attached kinetochores on sister chromatids. When the checkpoint requirement is satisfied after all sister chromatids are connected to the bipolar spindle, the inhibition towards APC/C–Cdc20 is diminished. APC/C–Cdc20 degrades NEK2A and cyclin A in prometaphase [54] and securin and cyclin B1 in metaphase. APC/C–Cdc20 complex binds to Cyclin B1 and commences its destruction when chromosome bi-orientation, which dependent on the spindle checkpoint. Recently studies revealed that MASTL is crucial for the recruitment of cyclin B1 to the APC/C, without the need of Cdc20, which subsequently results in CyclinB1 degradation once the checkpoint has been lifted [55, 56]. The ubiquitination and destruction of Securin, which is an inhibitor of Separase, led Separase to cleave the cohesin complexes and subsequently trigger sister chromatid segregation [57]. During metaphase, the spindle checkpoint is silenced and Cdk1 activity is minimized, which eventually gives a “GO” signal for anaphase to commence. In adverse to early-destroyed cyclins such as Cyclins A and B1, which restrain APC/C function, the distinct member of cyclin B family member, Cyclin B3 is a mitotic cyclin stimulates APC/C activity and promotes the metaphase–anaphase transition [58].

It is thought that Cdh1 maintains in silence from G2 and early mitosis due to its phosphorylation [30, 52]. At mitotic exit, including anaphase and telophase, dephosphorylation of Cdh1 by CDC14 [59], APC/C–Cdh1 is activated and ubiquitylates Cdc20 [60], Aurora kinases (Aurora A and B) [61, 62], Tpx2 [63] and Polo-like kinase 1 (PLK1) [64] which ensures a low kinase activity environment to pave the road for mitotic exit. Interestingly, it was recently reported that the APC/C regulates spindle formation through promoting the degradation of four spindle-binding proteins Bard1, Hmmr, HURP and NuSAP [65]. Furthermore, the stress-activated kinase JNK [66], HEC1 [67] and EYA1 [68] were also identified to be ubiquitin substrates of APC/C–Cdh1 during the transition from mitosis to the G1 phase. During G1 phase, APC/C–Cdh1 destructs mitotic cyclins, Cdc25A [69], Skp2 [70], Ndd1 [71], USP37 [48] and Cks1 [70] to sustain low Cdk activity. In addition, APC/C–Cdh1 regulates the destruction of replication regulators including Geminin [72] and Cdc6 [73], RRM2 [74], Claspin [75] as well as its own E2, Ube2C and UbcH10 [76, 77], which leads to inactivation of APC/C–Cdh1 and resulting stabilization of Cyclin A. While APC/C–Cdh1 destructs substrates during G1 phase specifically, these substrates are degraded via SCF ligases in other phase of the cell cycle. For examples, the substrates Cdc25A [78], Claspin [79], and USP37 [80] are destructed by SCF^β-TrCP^ in S and G2 phase; the substrate RRM2 is degraded via SCF^Cyclin F^ [81]. During G1–S transition, APC/C–Cdh1 is deactivated via two mechanisms: binding to Emi1 and degradation of UbcH10, UBE2C [76, 77]. During normal S and G2 phase, Cyclin A/Cdk2 binds and phosphorylates Cdh1 to uphold the APC/C in its deactivated state [82].

**Fig. 5:**
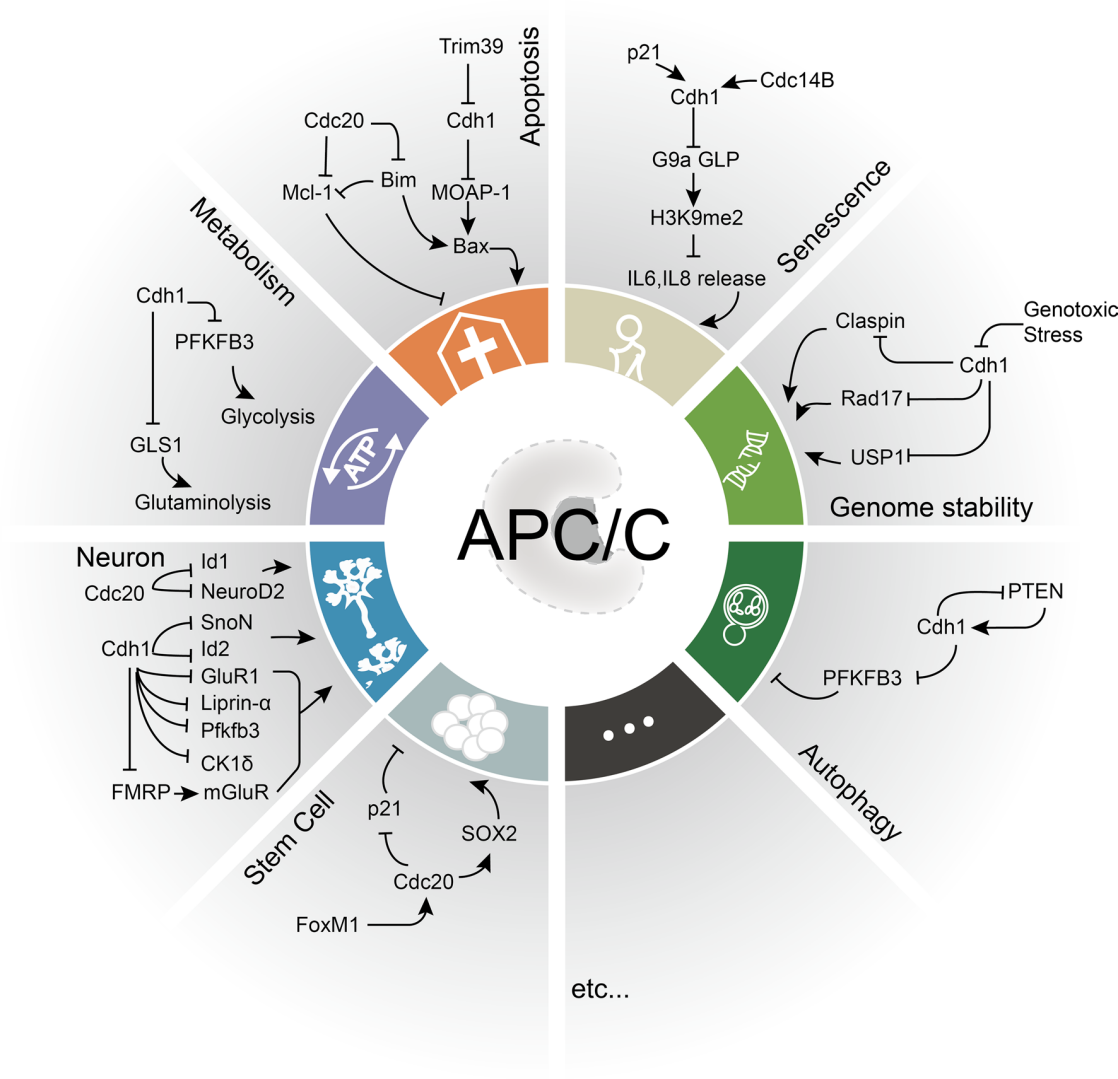
APC/C in genomic integrity, apoptosis, autophagy, senescence, metabolism, stem cell and neuron regulation. The up panel shows APC/C controls several process including genomic integrity, apoptosis, autophagy, senescence, metabolism, stem cell and neuron regulation. In genomic integrity regulation part, genotoxic stress induced APC/C–Cdh1 activation and subsequently ubiquitylates substrates Rad17, Claspin and USP1 to regulate cell cycle checkpoint and recovery. In senescence process, APC/C–Cdh1 is activated by CDC14B and p21 to ubiquitylate substrates G9a and GLP and subsequently provokes IL-6 and IL-8 transcription. In apoptosis panel, Cdh1 targets MOAP1/Bax and Cdc20 targets Mcl1 and Bim1 to control apoptosis process. In metabolism regulation, APC/C–Cdh1 targets PFKFB3 and GLS1 to control glycolysis and glutaminolysis. In neuron, APC/C–Cdh1-mediates degradation of fragile X syndrome protein (FMRP), CK1δ, GluR1, Liprin-α, and Pfkfb3, APC/C–Cdc20 mediates ubiquitylation of Id2 and SnoN. In Stem cell, APC/C–Cdc20 mediates degradation of p21 and regulates pluripotency-related transcription factor SOX2 protein transcription activity. The down panel shows how APC/C potential controls autophagy process. APC/C–Cdh1-mediates degradation of PFKFB3, a critical factor in glucose metabolism and induces autophagy. Loss of PTEN, reduces APC/C–Cdh1-mediated degradation of PFKFB3, lead to strong inhibition of autophagy. On the other hand, APC/C–Cdh1 mediated chromatin accumulated PTEN degradation during mitotic exit

Despite the master regulatory role in regulating cell cycle, accumulating evidence uncover the impact of APC/C in an assortment of cellular processes beyond cell cycle, including regulation of cell differentiation, genomic integrity, developmental processes and the nervous system (Fig. 5) [83, 84]. Many regulators of DNA damage repair and genomic stability such as CtIP [85], Claspin [86], UPS1 [87] and Rad17 [84] were recently characterized as bona fide Cdh1 substrates. Furthermore, the identification of Mcl-1 [88] and Bim [89] as a Cdc20 substrate as well as G9a and GLP [90] as Cdh1 substrates expands APC/C functionality into regulating cellular apoptosis and senescence. In addition, APC/C also participates in other cell cycle-independent functions including regulating cellular metabolism, cell mobility and gene transcription through degradation of specific substrates. Nevertheless, how APC/C–Cdh1 and APC/C–Cdc20 are regulated and recruited by various physiological signaling remains not very clear. Given involvement of multiple types of protein posttranslational modifications in response to signaling, if crosstalk between APC/C with other types of modifications in governing signaling module needs to be further studied.

## APC/C in genome stability

The major function of APC/C in regulating mitosis and meiosis is through dictating temporal chromatid segregation that ensures the fidelity of daughter genome. The segregation errors due to malfunction of APC/C activity leads to chromosomal instability (CIN) with deleterious consequences. Recently, genetic mouse model works specified the loss of Cdh1 related to centrosome amplification, chromosome missegregation, thus causing tumorigenesis [91]. Loss of the Cdh1 deacetylase SIRT2 leads to APC/C activity decrease and subsequential mitotic catastrophe, genetic instability, and tumorigenesis [34]. MCC complex member BubR1 [92], Bub3 [93, 94], Bub1 [95], MAD2 [96] haploinsufficiency causes APC/C–Cdc20 abnormal activated and lead to premature anaphase and loss of chromosome integrity in mammalian cells Moreover, overexpression of Bub1 [95], Mad2 [97] in transgenic mice have the consequences of broken chromosomes, anaphase bridges, chromosome gains and deletions, and increased rate of tumorigenesis. On the contrary, increased expression of BubR1 in transgenic mice seems to have a protective effect against aneuploidy and cancer [98]. Therefore, APC/C is critical for genomic integrity by regulating high fidelity mitosis, abnormal APC/C activity leads to genomic instability.

In recent year there has been considerable work to elucidate the mechanism of APC/C–Cdh1 and how it is able to control the DNA damage checkpoint response and DNA repair via degradation of substrates such as CtIP [85], Claspin [86], UPS1 [87] and Rad17 [84]. In response to DNA damage, APC/C–Cdh1 is activated by dephosphorylation by nucleolus–nucleoplasm translocated CDC14B as well as p53- and p21-dependent CDK1 inactivation and Emi1 downregulation [75, 99]. The APC/C–Cdh1 complex seems to regulate the DNA damage-induced G2/M cell cycle checkpoint. While PLK1 [75], MEF2C [100], FoxM1 [101], Wip1 phosphatase [102] are destructed by activated APC/C–Cdh1, other substrates like Claspin, 53BP1, Chk2 [103, 104] are protected. The APC/C–Cdh1 has also been involved in moderating DNA repair. After DNA repair, the DNA damage response and DNA repair machinery need to be shut down. In the DNA damage response as well as during exit from mitosis, CtIP was down-regulated by APC/C–Cdh1 [85]. Also during mitotic exit, the Rap80 complexes with BRCA1 to facilitate homologous recombination, and it is then subsequently degraded by APC/C-which seems to prevent non-regulated recombination during G1 [105]. Thus, it is well-known that correct activation of APC/C–Cdh1 is needed for robust DNA repair mechanisms, which has been shown with studies down in Cdc14B knockout cells in which its loss leads to no activation of APC/C–Cdh1 thus infective DNA repair [106].

The APC/C–Cdh1 has also been shown to regulate cellular replication in studies involving the deubiquitinating enzyme USP1 in which it was ubiquitinated and degraded by APC/C–Cdh1 allowing PCNA to be mono-ubiquitinated in response to UV [87]. It has been well known that the ATR–Rad17 cascade in which Rad9–Rad1–Hus1 is loaded on DNA with subsequent activation of Claspin/Chk1 is needed to activate cell cycle checkpoint [107]. UV exposure has also been shown to degrade Rad17 via APC/C–Cdh1, which seems to be required for entry back into the cell cycle [84]. The decrease of Cdh1 lead to Rad17 accumulation and tumorigenesis, which consistent with the Cdh1 deficiency mouse model [108].

## APC/C in apoptosis and senescence

Coordination between survival and death after cellular challenge from stress such as radiation shock or the treatment with chemotherapeutic drug determines the cellular fate. Recent studies implicate the impact of APC/C in apoptosis regulation. The APC/C–Cdc20 destructs anti-apoptotic Mcl-1 [88] and pro-apoptotic protein Bim [89]. CDK1/cyclin B1 phosphorylates Mcl-1 at two specific residues, Thr92 and Ser64. Phosphorylation of Thr92 starts Mcl-1 ubiquitination and degradation when cells stopped in mitosis. Therefore, Thr92 phosphorylation of Mcl-1 by CDK1 and its ubiquitination and degradation by APC/C–Cdc20 are implicated in the beginning of apoptosis in the event that a cell fails to undertake mitosis. Bim, a powerful pro-apoptotic factor, is also a substrate of APC/C–Cdc20. When prolonged inhibition of APC/C–Cdc20 using drugs that stabilize or depolymerize microtubules (Taxol and Nocodazole, respectively) induce mitotic arrest and Bim stabilization, which leads to cell apoptosis. Another pro-apoptotic Bcl-2 family members Bax is regulated by APC/C–Cdh1 mediated modulator of apoptosis protein 1 (MOAP-1) degradation [109, 110]. MOAP-1 is a Bax activation enhancer induced by DNA damage. APC/C–Cdh1-mediated MOAP-1 degradation is reversed by the ubiquitin ligase Trim39. The correlation between mitotic APC/C with apoptosis implicates the APC/C complex in being able to distinguish between normal events of mitosis and those that are prolonged events of mitotic arrest.

Senescence is a stage when growth has been suspended and is a critical barrier for tumors in vivo and it is well known that the DNA damage response machinery plays a crucial role in executing these specific phenotypes. Recently studies revealed the APC/C could control the senescence process. Takahashi et al. reported that DNA damage provokes primary Lys 9 of histone H3 mono- (H3K9me1) and demethylation (H3K9me2) transferases G9a and GLP degradation via APC/C–CDH1 that activated via Cdc14B- and p21^Waf1/Cip1^ [90]. The decrease of G9a and GLP lead to reduce H3K9me1/2 driving the transcription of senescence-associated secretory phenotype (SASP) interleukins IL-6 and IL-8 in senescent cells. Therefore, the APC/C–Cdh1–G9a/GLP signals axis links the DNA damage response (DDR) and SASP responses in senescent cells. Johmura et al. found that normal human diploid fibroblasts (HDFs) that were influenced by stimuli to bring about senescence interestingly underwent a skip in events of mitosis before entry permanent cell-cycle arrest which is mediated by p53 activation of APC/C–Cdh1 and pRb [111]. Activation of p53/p21 at G2 phase results in the impulsive activation of APC/C–Cdh1 that destroys various mitotic regulators, subsequently leading a switch in roles of Cdt1 and inducing senescence.

## APC/C in autophagy

One of the newest findings in recent years is the connection between APC/C with autophagy. Autophagy is a natural regulated degrading mechanism that regulated the coordinated degradation and recycling of cellular compounds [112]. During the events of autophagy, it is well known that autophagosomes are created which then subsequently fuse with a lysosomal organ and the internal components are then degraded via lysosomal enzymes. Autophagy has been implicated as a mechanistic consequence of stress promoting cell survival, however in different scenarios, it has been shown to promote cell death [112]. Studies have shown the association of APC and Cdh1 plays important role in regulating autophagic process bifunctional 6-phosphofructo-2-kinase/fructose-2,6-bisphosphatases (PFKFBs) controls glycolysis by regulating the levels of fructose 2,6 bisphosphate (F2,6BP), a critical activator of phosphofructokinase 1 (PFK-1). The PFKFB family comprise four isoforms of which PFKFB3 is of specific concern to the pharmaceutical industry since PFKFB3 mRNA has been shown to be elevated in certain tumors. Recently study demonstrated loss of PTEN, a well-known tumor suppressor, reduces stabilization of PFKFB3 by enhancing APC/C–Cdh1-mediated degradation [113]. Inhibition of the PFKFB3 decreases cancer cell glucose metabolism and induces autophagy [114, 115]. This consists with the reports that loss of PTEN, causes the strong inhibition of autophagy [116–118]. On the other hand, phosphorylated PTEN by PLK1 accumulates on chromatin during mitosis, and the APC/C–Cdh1 facilitate removal of chromatin-bound PTEN, which is a critical step for mitotic exit [119, 120]. In addition, in response to stress resulting from protein damage, APC/C is responsible for the ubiquitination and subsequent degradation of heat shock factor 2 (HSF2). HSF1 and HSF2 are transcription factors contributing expression of heat shock proteins (Hsps) by directly binding to the Hsp70 promoter in response to stress [121]. HSF2 was also suggested induce autophagic cell death upon heat shock [122]. This indicated the APC/C–Cdh1 could be a critical regulator in autophagy onset by degrading PFKFB3 and HSF2. While the reports on the observation of APC/C in regulating autophagy is still increasing, the detailed mechanism by which how APC/C is recruited to modulate the process of autophagy and its working mechanisms both in vitro and in vivo are needed to be addressed carefully.

In addition to APC/C potential control autophagy process, autophagy could in reverse regulate APC/C activity. Dotiwala et al. reported that in budding yeast hyperactivation autophagy induced by DNA damage, causes nuclear exclusion of both esp1/separase and Pds1/securin, which counteract the nuclear degradation of Pds1 by APC/C, and leads to a permanent G2/M arrest of cell [123]. Glucose withdrawal can decrease levels of ATP which then subsequently begin an autophagy cascade to increase levels of ATP via lysosomal degradation [124, 125]. The APC/C activation also depends on the hydrolysable ATP and needs chaperone ATPase such as HSP70 and HSP90 [126, 127]. Recent studies revealed that HSP70 inhibitor PES-Cl inhibit both autophagy and the activity of APC/C and lead to cell cycle arrest, which indicates HSP70 may bridge the autophagy and APC/C activity regulation [128].

## APC/C in metabolism

Emerging observation in has implicated APC/C in regulating cellular metabolism. In brain energy metabolism, cortical neurons actually have a decreased capacity to utilize glucose via glycolysis compared to the metabolism of astrocytes, instead they utilize glucose to maintain regulated levels of antioxidants via APC/C–Cdh1/Pfkfb3 [129]. PFKFB3, which is a rate-limiting regulator of glycolysis through the generation of fructose-2,6-bisphosphate (F2,6BP), was initially reported to be degraded by APC/C–Cdh1 in neurons [130]. In astrocytes, PFKFB3 is constantly existent due to low APC/C–Cdh1 activity, but in neurons, the PFKFB3 is absent that it is always subject to proteasomal degradation by APC/C–Cdh1. In brain energy metabolism, APC/C–Cdh1 activity is repressed by over-activation of glutamate receptors NMDAR (*N*-methyl-d-aspartate subtype of glutamate receptors) through Ca^2+^-Cdk5-dependent signaling pathway which leads to Cdh1 phosphorylation [131]. Activation of NMDAR by glutamate analog NMDA caused PFKFB3 stabilization leading to increased glycolysis and reduced action of the pentose-phosphate pathway (PPP) which triggered oxidative stress and resulting neuronal death by excitotoxicity. Therefore, targeting the NMDAR-APC/C–Cdh1/PFKFB3 protein regulation cascade which controlling brain energy metabolism is potential a novel therapeutic strategy for neurodegenerative diseases.

It has demonstrated that metabolism and cell cycle progression are integrated and coupled with each other. The initiation step of metabolic machinery adaptation includes Glycolysis and glutaminolysis. In the process of cellular division, the activation of glycolysis and glutaminolysis are strict via the effects of two ubiquitin ligases, APC/C–Cdh1 and SCF^β-TrCP^ which regulate the stabilization and activity of PFKFB3 and Glutaminase 1 (GLS1) [132]. Colombo et al. [133] report that the diminishing in APC/C–Cdh1 activity that occurs in late G1 phase leads to the accumulation of PFKFB3 and GLS1, and, subsequently, raised glycolysis and glutaminolysis to frustrate the restrictive checkpoint of the G1 phase by keeping up elevated amounts of glycolytic and glutaminolytic intermediates. These studies are consistent with previous finding that overexpression of Cdh1 largely averts the increase in glycolysis and glutaminolysis and reduces S phase cells proportion [134]. Interestingly the levels of PFKFB3 and GLS1 both are already low expression in G2 phase, when APC/C–Cdh1 is still inactive, implying the involvement of extra regulatory mechanisms, which subsequently shows how this restructuring of regulatory machinery is crucial to cellular proliferation.

## APC/C in stem cells

Maintenance of stem cell self-renewal and regulation of stem cell terminal differentiation are critically correlated with cell-cycle control that orchestrates tissue specification, organ homeostasis, and potentially tumorigenesis [135]. As a master mitotic regulator, APC/C is supposed to have an essential role in regulating the self-renewal and differentiation of stem cells. In drosophila, the Cdc20/fizzy (fzy) has been suggested to suppress catastrophic cellular stress induced necrosis in neural stem cells [136]. Loss of Cdc20/fizzy in neuroblasts showing reduced APC/C activity resulting necrosis, by contrast, the gain of non-degradable type of APC/C substrates required for cell cycle progression leads to mitotic defect. Mao et al. [137] reported that APC/C–Cdc20 controls human glioblastoma stem-like cells (GSCs) invasion and self-renewal, and its tumorigenicity in vivo. APC/C–Cdc20 interacts with and regulates pluripotency-related transcription factor SOX2 protein transcription activity but not degradation and results driving GSC invasiveness and self-renewal. During prometaphase, APC/C–Cdc20 mediating the degradation of p21 leads to full activate CDK1 and prevents mitotic slippage [138]. Xie et al. [139] reported that APC/C–Cdc20, which is transcriptionally controlled by Forkhead transcription factor M1 (FoxM1), maintains tumor initiating cells (TICs) through degradation of p21CIP1/WAF1, a critical negative regulator of TICs in glioblastoma. FoxM1 activity is needed for the appropriate expression of different types of controller of mitosis, such as Cyclin B, Plk1, Aurora B and Cdc25B [140]. Interestingly, the foxM1 is also a substrate of APC/C–Cdh1 [101, 141]. FoxM1 is degraded at mitotic exit by APC/C–Cdh1 and its degradation is critical for regulated entry into S phase. Therefore, APC/C–Cdh1 may have different role in tumor initiating cells regulation.

In embryonic stem cells (ESCs), the cell cycle features show comparative S phase to somatic cells but have unusually shorter G1 and G2 phase [142]. The APC/C substrates such as Aurora A, Cdh1, Cdt1, Cyclin A, Cyclin B, Geminin, Plk1, and Securin decrease significantly after mitotic exit, but the degradation of the substrates is not as significant as that seen in somatic cells [143]. APC/C–Cdh1 is inactive in undifferentiated ESCs but do become active as levels of inhibitors of Emi1 decrease [144]. Also unlike somatic cells, in the G1 and S phases it has been shown that Cdc20 is present but dissociates from APC/C of ESCs cells [143]. Mice with no Cdc20 function proved to be embryologically fatal due to cell cycle arrest in metaphase at the two-cell stage with high levels of cyclin B1 [145].

## APC/C in neuron

Accumulating evidence suggests that APC/C is critical in regulating neuron development and neuronal function via governing the turnover of some neuron-specific proteins. Specifically, APC/C–Cdh1 was found to control axon growth and patterning in the process of normal brain development [146]. Subsequent studies reported that mechanistically, APC/C–Cdh1 regulates neuronal development through targeting two axon growth-promoting factors, Id2 and SnoN, for degradation [147, 148]. Subsequent studies revealed that APC/C–Cdc20 regulates dendrite morphogenesis and presynaptic differentiation through degradation of the transcription factors Id1 and NeuroD2 [149, 150]. Further studies showed that synaptic plasticity, synaptic size and the bioenergetics and antioxidant status of neurons are controlled by APC/C–Cdh1 mediated degradation of GluR1 [151], Liprin-α [152, 153] and Pfkfb3 [113]. Recently studies revealed APC/C–Cdh1 driving the hippocampal mGluR-dependent synaptic plasticity in the mammalian brain through degrade the fragile X syndrome protein (FMRP) [154]. APC/C–Cdh1 targets CK1δ for degradation which regulates cerebellar granule cell neurogenesis [155]. Although several aspects of how the APC/C regulates the nervous system have been uncovered at the cellular level, it remains largely unclear how at the organismal level, APC/C deficiency could affect neuronal function, including mammalian learning and memory [156], and whether APC/C functions in neurological and psychiatric disorders.

## APC/C in tumorigenesis

Most of APC/C regulated cellular functions are directly or indirectly connected to tumor initiation or invasion. Results from pathological studies unveiled a series of mutations in ANAPC3/Apc3, ANAPC6/Apc6, and ANAPC8/Apc8 in breast cancer, colon cancer, glioma, hepatocarcinoma, melanoma, neuroblastoma, choriocarcinoma tissues [157]. Further results based on mouse targeting deletion or xenograft studies demonstrated that APC/C coactivator Cdc20 or Cdh1 to be as oncoprotein or tumor suppressor in many types of cancer [83].

Recently, some studies have shown that increased Cdc20 expression is concomitant with clinical progression in various types of human tumors which consistent with the notion that Cdc20 may have functions similar to many oncoproteins. For example, high expression of Cdc20 was observed in primary non-small cell lung cancer (NSCLC) patients [158], colorectal cancer [159]. Moreover, Cdc20 expression was positively correlated with clinicopathological parameters including invasion, and pathological tumor status. In genetic mouse model, mouse loss of Cdc20 is embryonic lethality at the two-cell stage because of metaphase arrest [145]. Further studies revealed APC/C cofactor Cdc20 is crucial for anaphase onset in vivo in both embryonic cells and somatic cells including progenitor cells [160]. In a tamoxifen inducible conditional Cdc20 knockout mouse (Cdc20^−/lox^/RERT^+/Cre^) chemical induced skin cancer model, ablation of Cdc20 results in Cdc20 ablation can subsequently result in complete tumor regression in vivo via apoptosis. Further histological analysis revealed that depletion of Cdc20 in skin tumors resulted in tumor cell arrest in metaphase, accompanied by induction of cellular apoptosis [160].

Cdh1, an APC/C cofactor is a well-known tumor suppressor [161]. Through studies it has been shown that inhibition of Cdh1 can lead to centrosome amplification and incorrect chromosome segregation, and thus has been associated with genetic instability and tumorigenesis, Cdh1 deficient (Fzr1^+/−^) mice develop several types of epithelial tumors, such as fibroadenomas and mammary gland adenocarcinomas, which are not observed in Cdh1 wild-type (Fzr1^+/+^) mice [91]. Notably, Cdh1 expression has been shown to be decreased in ovary, prostate, breast, colon, brain and liver tumor cell lines [75, 108, 162, 163]. Concomitant with downregulation of Cdh1 expression, several APC/C–Cdh1 targets, such as Aurora A, Aurora B, Cdc6, Cdc20, Cyclin B, Rad17 and Tpx2 are often upregulated in human cancer tissue samples [164]. On the other hand, Lehman et al. demonstrated that Cdh1 was overexpressed in certain tumor types [165].

## APC/C and drug development

**Fig. 6:**
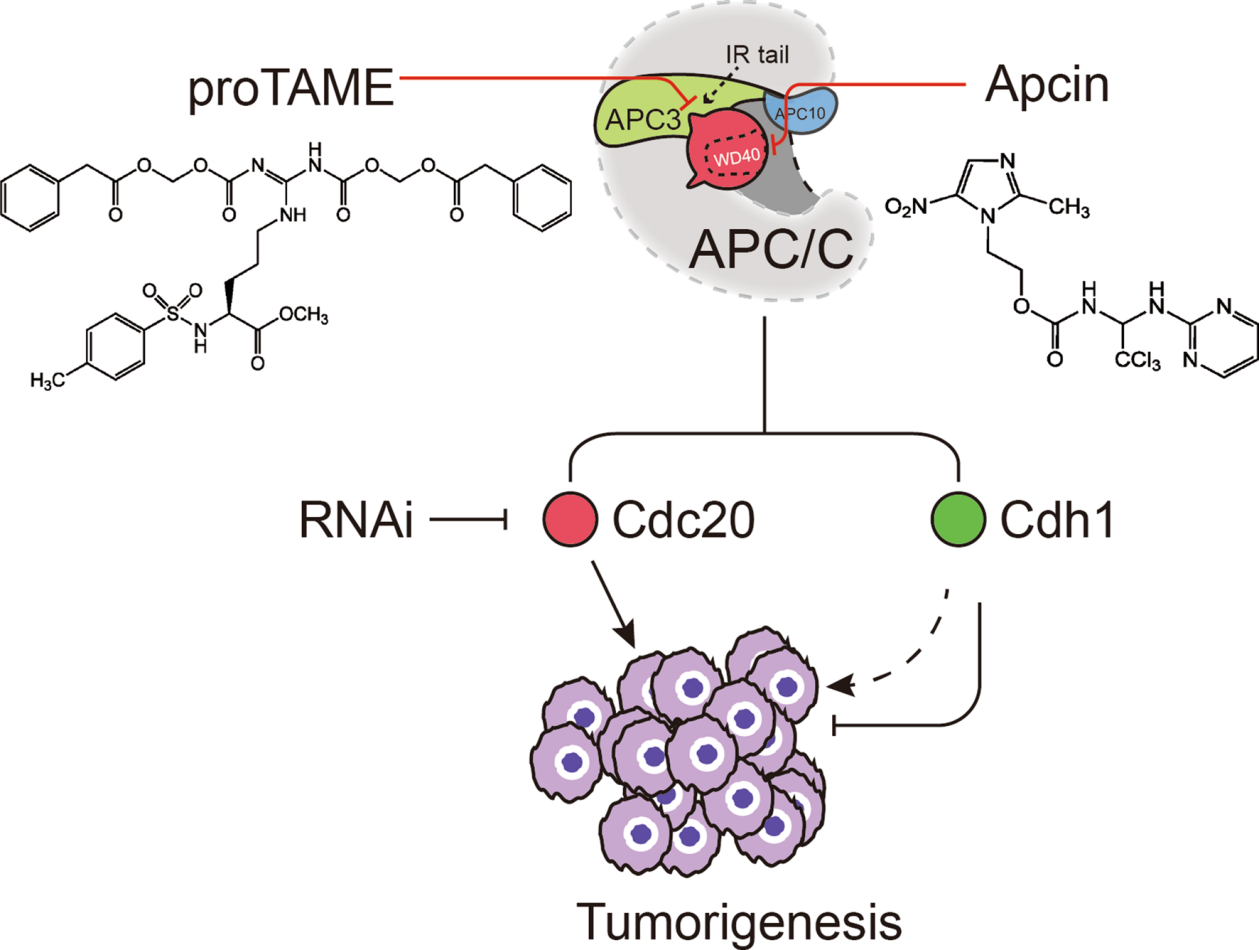
APC/C in tumorigenesis and drug development. Currently, Cdc20 has been recognized as tumor enhancer, whereas Cdh1 has been recognized as tumor suppressor in most type of cancer. Developing specific APC/C inhibitors are potential therapeutic target for cancer treatment. Two inhibitors have been developed, the pro-TAME disrupt APC3-Cdc20 IR-tail binding and Apcin inhibit the D-box binding between Cdc20 and the substrate. The pro-TAME and Apcin have synergistic blockade of mitotic exit effect due to different APC/C activity disrupt mechanism

Given the pivotal role of APC–Cdc20 in governing mitotic progression, blockade of chromatid segregation or mitotic exit largely attracts the attention for development of small molecule inhibitor that could be utilized to suppress cancer cell growth or induce cancer death. In recent studies, it was shown that a relevant target might be mitotic exit because it the pro-apoptotic consequences of RNAi against the APC/C cofactor, Cdc20 [166]. Several APC/C inhibitors have been developed recent years, such as pro-TAME [167], Apcin [168] (Fig. 6). The pro-TAME disrupts APC3–Cdc20 IR-tail binding interaction but in this case, Cdc20 can still be enlisted to the APC/C via the interactions between its C-box and co-receptors. TAME discharges Cdc20 from the APC/C by boosting Cdc20 auto-ubiquitination and subsequent Cyclin B1 stabilization [169]. It was also shown that Cyclin B1 counteracts TAME’s effect by boosting recruiting of free Cdc20 to the APC/C, meanwhile, Cdc20 autoubiquitination is also decreased. Most recently, Sackton et al. revealed combined use of Apcin, which disrupts D-box interaction between Cdc20 and the substrate, and TAME jointly disrupt the interface between APC/C, Cdc20 and substrate and thereby having the combined effect of increasing the duration of mitosis and blocking its exit [168].

It has also been shown that use of anti-mitotic drugs could prove to be lethal with the use of chemical drugs. Giovinazzi et al. also reported proTAME prohibited mitotic exit of paclitaxel and Aurora A inhibitor MLN8054 arrested cells induced apoptosis [170]. Eguren et al. reported that loss of Cdh1 results in an increased sensitivity to DNA topoisomerase 2-alpha (Top2α) inhibitors such as etoposide and ICRF-193 as an outcome of augmented amount of Top2-DNA trapped complexes [171]. It is also worth noting that in cancer cells inhibition of APC/C by chemical inhibitor pro-TAME could sensitize Top2α inhibitors. While the results of validation of the above inhibitors based on cultured-cell and xenograft models shed light on novel anti-cancer strategy, preclinical study with combination to various chemo drugs in different patient-derived xenograft models will further validate and enhance the potential of APC inhibitors in future’s anti-cancer treatment.

## Conclusion and future direction

Although APC/C was initially characterized to be a master regulator of cell cycle control, results from over decade uncovered its feature as a multiple functional ubiquitin protein ligases. Demonstration of its participation in various types of cellular processes, environmental stress as well as communication between pathogen/host largely implicates its impact in the maintenance of homeostasis and otherwise diseases. Recently years’ progress in 3D structural studies and different types of ubiquitin chains catalyzed by APC/C significantly enhance the in-depth view about how the APC/C works and its regulation. Development of its small molecule inhibitors shed light on its potential value in anti-cancer treatment and other diseases. While novel function of APC/C is linked to regulation of cellular metabolism, emerging evidence has also sketched the previous undocumented role to autophagy. Despite the exciting new findings for APC/C, following future efforts could further enhance our understanding of APC/C and promote its translational value in anti-cancer treatment. Current 3D structural work from Barford and other laboratories provides much clear and accurate topological picture for APC/C subunits as well as the activators, which allows a better designing of more potent small molecule modulators for chemical genetic study and drug development. Information based on the findings of various types of chains catalyzed by APC/C would encourage more detailed work from the view of physiology, which could validate the relevance of fine-tuning from the level of ubiquitin chain formation. Biochemically, how exactly the large complex of APC/C is assembled still remains unclear. Some recent results based on mass spectrometry analyses suggest that one functional protein could be simultaneously regulated by multiple-types of posttranslational modifications in order to achieve certain physiological effect. Thus, if APC/C-mediated ubiquitylation interplays with other posttranslational modification in orchestrating substrate protein in response to signaling or stress needs to be considered. Beyond cell cycle control, if APC/C-mediated catalysis needs entire 15 subunits or whether smaller complex with necessary subunits could satisfy the commitment for APC/C under various physiological circumstances is unknown. Several deubiquitinases were reported to be involved in APC/C governed regulation by counteracting its ubiquitylation. Given the large list of APC/C substrates, if certain deubiquitinases could be shared by different APC/C regulated substrates remain unknown. It’s now clear that APC/C has a critical responsibility in cell division, stem cell regulation, neuronal processes, cell death and tumorigenesis. Besides the basic research, it is anticipated that further validation of newly developed APC/C small molecule inhibitors by utilizing various animal disease models would lead to a new era for APC/C.

## Abbreviations

9-1-1: Rad9–Rad1–Hus1
APC/C: anaphase promoting complex/cyclosome
Bub3: budding uninhibited by benzimidazole protein 3
BubR1: bub1-related protein
Cdc20: cell division cycle 20
Cdh1: Cdc20 homologue protein 1
CIN: chromosomal instability
DDR: DNA damage response
DUBs: deubiquitinases
Emi1: early mitotic inhibitor 1
Emi2: early mitotic inhibitor 2
ESCs: embryonic stem cells
FMRP: fragile X syndrome protein
FoxM1: forkhead transcription factor M1
GLS1: glutaminase 1
HECT: homologous to the E6-AP carboxyl terminus
HSF2: heat shock factor 2
KO: knockout
LATS1: large tumor suppressor kinase 1
LATS2: large tumor suppressor kinase 2
Mad2: mitotic arrest deficient protein 2
MCC: mitotic checkpoint complex
MOAP-1: modulator of apoptosis protein 1
NMDAR: *N*-methyl-d-aspartate subtype of glutamate receptors
4-OHT: 4-hydroxytamoxifen
PFKFBs: 6-phosphofructo-2-kinase/fructose-2,6-bisphosphatases (PFKFBs)
PLK1: Polo-like kinase 1
PPP: pentose-phosphate pathway
RING: really interesting new gene
SAC: spindle assembly checkpoint
SASP: senescence-associated secretory phenotype
SCF: Skp1–Cullin-1–F-box protein
Skp1: S-phase-kinase-associated protein 1
β-TRCP: β-transducin repeat-containing protein
Ub: ubiquitin
UPS: ubiquitin–proteasome system
USP22: ubiquitin-specific protease 22
USP37: ubiquitin-specific protease 37
USP44: ubiquitin-specific protease 44
